# Construction and Application of an Intelligent Response System for COVID-19 Voice Consultation in China: A Retrospective Study

**DOI:** 10.3389/fmed.2021.781781

**Published:** 2021-11-23

**Authors:** Jinming Shi, Jinghong Gao, Yunkai Zhai, Ming Ye, Yaoen Lu, Xianying He, Fangfang Cui, Qianqian Ma, Jie Zhao

**Affiliations:** ^1^The First Affiliated Hospital of Zhengzhou University, Zhengzhou, China; ^2^National Engineering Laboratory for Internet Medical Systems and Applications, Zhengzhou, China; ^3^Management Engineering School, Zhengzhou University, Zhengzhou, China; ^4^Henan Province Telemedicine Center of China, National Telemedicine Center of China, Zhengzhou, China

**Keywords:** intelligent response system, voice consultation, construction, application, COVID-19, China

## Abstract

**Background:** The outbreak of novel coronavirus disease 2019 (COVID-19) has led to tremendous individuals visit medical institutions for healthcare services. Public gatherings and close contact in clinics and emergency departments may increase the exposure and cross-infection of COVID-19.

**Objectives:** The purpose of this study was to develop and deploy an intelligent response system for COVID-19 voice consultation, to provide suggestions of response measures based on actual information of users, and screen COVID-19 suspected cases.

**Methods:** Based on the requirements analysis of business, user, and function, the physical architecture, system architecture, and core algorithms are designed and implemented. The system operation process is designed according to guidance documents of the National Health Commission and the actual experience of prevention, diagnosis and treatment of COVID-19. Both qualitative (system construction) and quantitative (system application) data from the real-world healthcare service of the system were retrospectively collected and analyzed.

**Results:** The system realizes the functions, such as remote deployment and operations, fast operation procedure adjustment, and multi-dimensional statistical report capability. The performance of the machine-learning model used to develop the system is better than others, with the lowest Character Error Rate (CER) 8.13%. As of September 24, 2020, the system has received 12,264 times incoming calls and provided a total of 11,788 COVID-19-related consultation services for the public. Approximately 85.2% of the users are from Henan Province and followed by Beijing (2.5%). Of all the incoming calls, China Mobile contributes the largest proportion (66%), while China Unicom and China Telecom are accounted for 23% and 11%. For the time that users access the system, there is a peak period in the morning (08:00–10:00) and afternoon (14:00–16:00), respectively.

**Conclusions:** The intelligent response system has achieved appreciable practical implementation effects. Our findings reveal that the provision of inquiry services through an intelligent voice consultation system may play a role in optimizing the allocation of healthcare resources, improving the efficiency of medical services, saving medical expenses, and protecting vulnerable groups.

## Introduction

In late December 2019, a cluster of pneumonia cases caused by a new severe acute respiratory syndrome coronavirus-2 (SARS-CoV-2) were firstly reported in Wuhan, Hubei Province, China (1, 2). The viral pneumonia was now officially known as novel coronavirus disease 2019 (COVID-19), which has been confirmed with the characteristic of human-to-human transmission (1, 3, 4). As of February 14, 2021, COVID-19 has extended to almost all of the countries or territories around the world, causing over 108.2 million confirmed COVID-19 patients that includes more than 2.3 million deaths (5). The COVID-19 pandemic has led to a large number of healthy, suspected, or asymptomatic-infected individuals visit medical institutions for diagnosis or treatment, resulting in a shortage of healthcare resources, and lots of people crowd in clinics and emergency departments (6, 7). The public gatherings may increase the infection risk of healthy people and medical staff. The COVID-19 outbreak has also caused a sharp increase in the demand for healthcare consultation services, far exceeding the capacity that medical institutions can bear (8, 9). In terms of the issues, it is necessary to carry out education, publicity, teleconsultation, and intelligent voice inquiry to assist people to take suitable prevention and control actions, such as fever clinic visits, quarantine, or self-isolation observation at home, respectively, according to their actual situations (10–13).

Given the situation of the COVID-19 pandemic, avoiding tremendous population visits to medical institutions and realizing remote healthcare consultation and triage of patients have become the important means to allocate healthcare resources optimally, which can contribute to the control of the pandemic indirectly. To date, one of the imperative tools that have not yet been fully explored is to employ information and communications technologies (ICTs) to support social distancing and quarantine, optimal healthcare delivery, and reduction of exposure and cross-infection for healthcare professionals and COVID-19 patients (14, 15). Intelligent conversational agents and virtual assistants, such as chatbots, wearable devices, voice assistants, and mobile phone applications, have proven their potential to serve as an intermediary in the fight against COVID-19 (14, 16). For example, during the COVID-19 pandemic, emerging voice assistants (e.g., Google Assistant, Apple Siri, and Amazon Alexa) have been adopted as an alternative healthcare delivery modality to mitigate the risk of COVID-19 spread and relieve the stress on the healthcare system (14, 15, 17).

According to the analysis of voice messages of users, an artificial intelligence-based voice consultation system for COVID-19 can automatically identify health consultation questions of users from different distances and then provide specific answers and response opinions (18–20). As the latest application of ICTs, the design and application of intelligent response systems for COVID-19 voice consultation have specific requirements for the development and deployment of relevant information systems (20, 21). Firstly, intelligent speech recognition based on machine learning needs to be accurate enough, and it can continuously self-learn and optimize as new data are imported. Secondly, data transmission quality, request-response speed, and load-carrying capacity of the information system should meet the actual needs of medical services. Thirdly, the system is able to support the full access of mobile phones, computers, and other terminals. Lastly, the system should set up a special security module to protect privacy of users and ensure information security.

Accordingly, to reduce the number of non-essential in-person visits at hospitals, lessening face-to-face contact among the healthy public, COVID-19 patients, and healthcare professionals, preserving already strained medical resources, increasing service capacity of medical institutions to screen suspected cases and deliver healthcare information, and eventually help reduce the spread of COVID-19, this study has developed and deployed an intelligent response system for COVID-19 voice consultation. The system would support the functions of collecting and analyzing information of users through intelligent inquiry and interaction with users, propose suggestions of corresponding mitigation measures based on the actual situations of the users, and screen suspected COVID-19 cases. According to our knowledge, this intelligent response system is one of the first tools developed and applied on a large scale for COVID-19 in China. The findings of the present study may play a helpful role in avoiding the frequent visits of healthy people to fever clinics, improving the utilization efficiency of medical resources, and preventing and controlling the COVID-19 infection among healthy people and medical practitioners.

## Materials and Methods

### Requirements Analysis

This study intends to design an intelligent response system for COVID-19 voice consultation, which can complete user self-assessment. When users access the system, the system will ask questions about the basic information of users. Through natural language processing (NLP) technology, it dynamically adjusts the follow-up questions that need to be confirmed according to the different options selected by the user and then give corresponding response opinions depending on actual situations, which can quickly screen out COVID-19 suspected cases and offer specific suggestions for further actions. Due to the incompleteness of online consultation, the system is not connected to any healthcare systems, once a COVID-19 case is suspected, she/he will be recommended to go to the nearest COVID-19 designated hospital for further confirmation and treatment immediately. To achieve the above functions, according to the requirements analysis of software engineering, the intelligent response system needs to meet the following items.

1. Business requirements: Build an intelligent voice consultation system for COVID-19, users can complete self-assessment by accessing the system. For related workers, the chances of contact with other people should be minimized during the development, deployment, and application stages of the system.
2. User requirements: Users only need to make a phone call to access the system. They can complete a self-assessment about COVID-19 without going to the hospital. Through real-time voice communication, individualized assessment results and suggested response measures are ultimately obtained.
3. Functional requirements:
  a) Remote deployment and maintenance: Given the high infectiousness of COVID-19, non-contact should be the first requirement of this system, and remote operation should be achievable at all stages from deployment to application of the system.
  b) Accurate speech understanding: Accurate and real-time speech understanding is a key to the successful application of this system. Minor mistakes in speech recognition and understanding may lead to severe errors.
  c) The intelligence of the system: Each epidemiological history and personal symptoms of user are different. Using a fixed process in all consultations is not appropriate, the system should be able to dynamically and intelligently adjust its contents based on the subsequent answers of user.
  d) Data statistics and analysis: Statistical analysis of data from system logs and routine operation can quickly identify characteristics of users and provide suggestions for improving the consultation framework, and it is also helpful for medical staff to conduct scientific research.

### Physical Architecture

**Figure 1:**
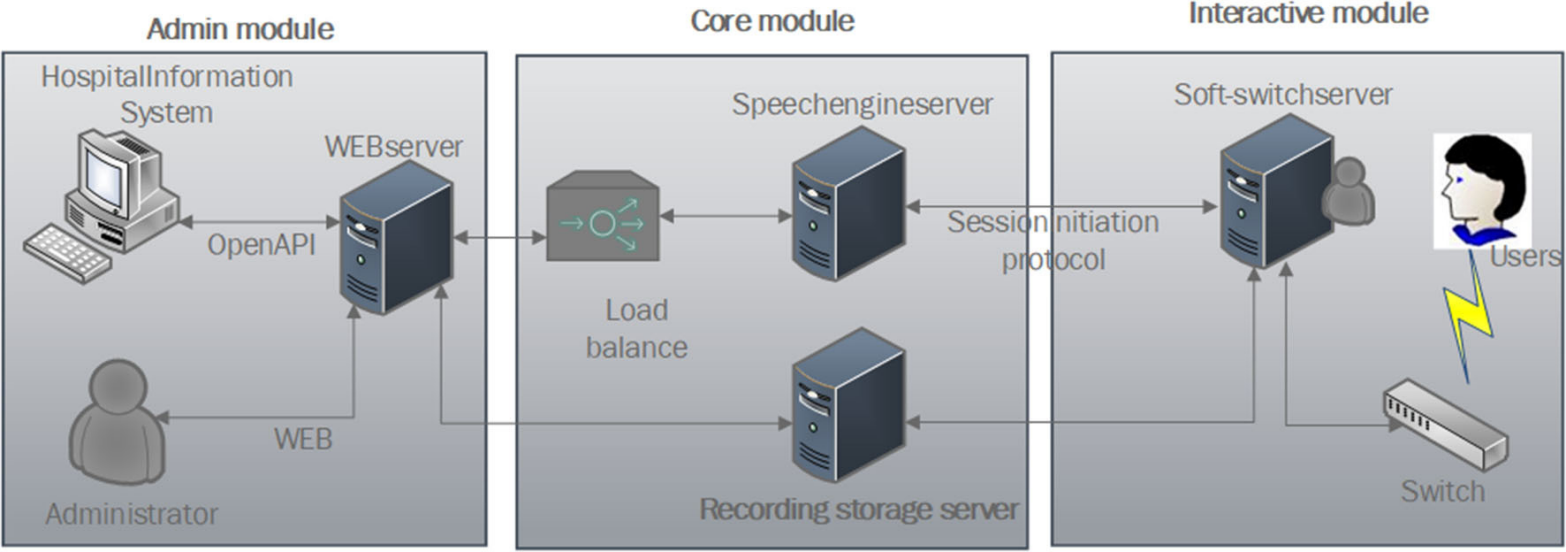
Physical architecture diagram.

To meet the abovementioned requirements, the physical architecture of the system is designed as shown in Figure 1. The physical architecture of the system consists of three modules, the administrator can adjust the operation procedure of system by admin module, and users can access the system by interactive module. The system acquires information, such as symptoms and epidemiological history of users, after establishing a call and collects the information provided by the user through a core module.

#### Admin Module

System administrators can access the system backstage remotely through web and then design and adjust the consultation process and configure the relevant parameters of the system. To realize subdivided medical services, the web server is open with various application programming interfaces (APIs) and can also contact with a hospital information system according to actual business needs.

#### Core Module

It contains three parts: intelligent voice engine server, load balancing, and storage server. The speech engine completes interactive services, such as speech recognition, synthesis, and understanding. The system dynamically balances services through load balancing, and the recording storage server is used to store recordings of the users.

#### Interactive Module

Users can directly dial the phone number to access the soft-switching system. The soft-switching system interacts with the core module through the Session Initiation Protocol and transmits the data of the conversation process to the recording storage server simultaneously.

### System Architecture

**Figure 2:**
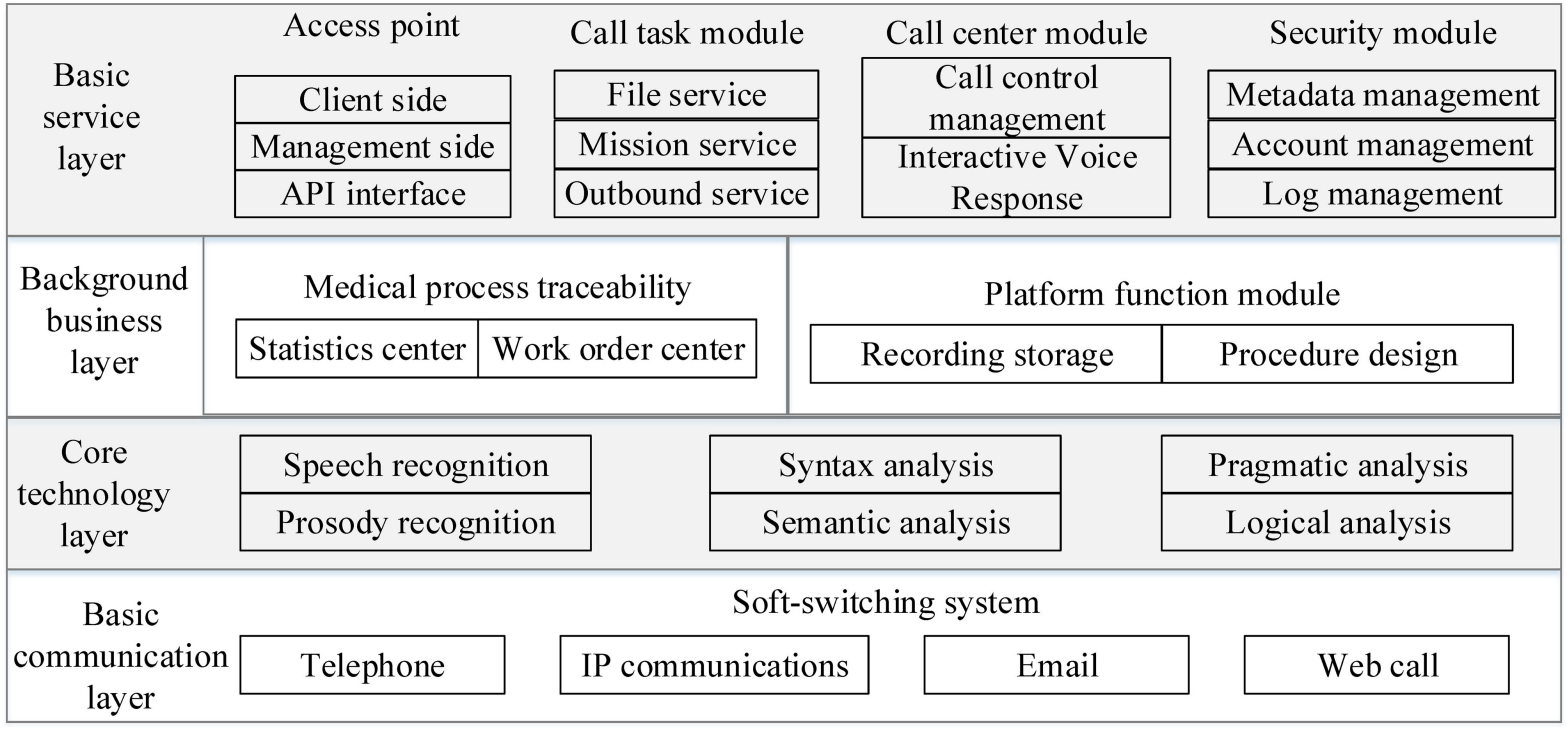
System architecture.

The architecture of the system is shown in Figure 2, which includes four layers.

#### Basic Communication Layer

This layer adopts a soft-switching system to handle different types of incoming calls. It supports multiple pathways of access, such as telephone, email, short messaging service (SMS), and Web call.

#### Core Technology Layer

This layer is the core layer of the system. Key technologies, such as automatic speech recognition (ASR), prosody recognition, pragmatic analysis, and syntax analysis are employed, which can work together to achieve multiple rounds of dialogue with the system.

#### Background Business Layer

The background business layer does not provide services directly to users, but it is open to system administrators to support related business.

#### Basic Service Layer

This layer is in charge of functions, such as user terminal management, outgoing and incoming call tasks management, call center control, API management, and security module, which provides services directly to users and engineers of system operation.

### Sampling Methods

Two samples were used in the present study. Firstly, sampling the voice signal, before training the model, it is necessary to sample the sound wave to convert the analog audio signal into a digital signal, which is convenient for the computer to process. This work was completed by using the following arguments: sample rate 8 kHz, bit depth 16 bit, and bit rate 128 kbps. After sampling the voice signal, Mel-scale frequency cepstral coefficients were employed to sample features of digital signals, which was able to achieve efficient modeling of the principles of the human voice, while reducing feature dimensions. Secondly, sampling the data set of speech recognition training. Before putting the speech recognition system into practical application, one of the biggest challenges is the accent problem. Actually, there are many accents in China, and the accents from different dialect regions are quite different (22). To deal with this problem, through cluster sampling, about 12,000 h of telephone voice conversation were collected from the company-provided voice communication service. The company is a leading voice service provider in China, with various customers from banks, hospitals, universities, and insurance companies, which ensures that the corpus includes various accents in different regions in China. The materials contain both human-to-human calls and human-to-machine calls. Thus, using the corpus to train and develop our models can effectively improve the accuracy of speech recognition and enhance the generalizability of the intelligent response system for COVID-19 voice consultation.

### Core Algorithms

**Figure 3:**
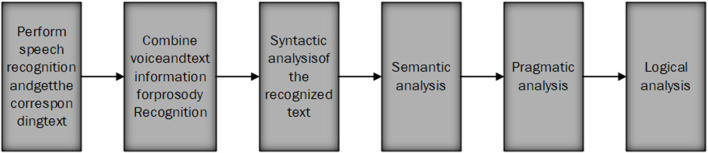
Algorithm flowchart.

The core algorithm of this system is based on multiple rounds of speech recognition. The implementation process of the algorithm is shown in Figure 3. Through speech recognition, prosody analysis, syntactic analysis, semantic analysis, pragmatic analysis, and logical judgment, multiple rounds of dialogue are realized.

#### Speech Recognition

**Figure 4:**
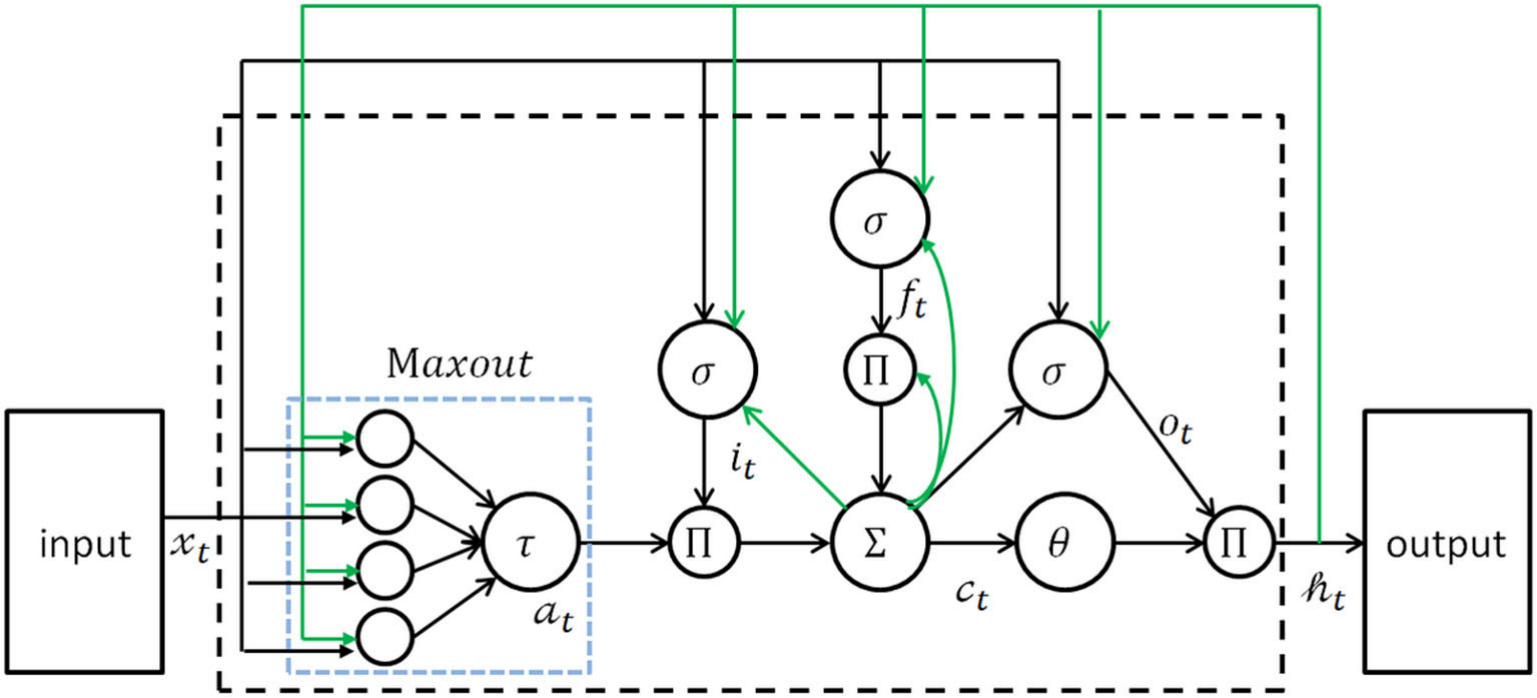
The architecture of maxout LSTM network. LSTM, long short-term memory.

Complete the conversion of speech to text by constructing a speech recognition acoustic model based on long short-term memory (LSTM). Since voice is a typical timing signal, a recurrent neural network (RNN) has strong timing modeling capabilities and therefore is suitable for voice recognition. The LSTM model is a variant of RNN, which has three more gates: forget gate, input gate, and output gate. Compared to traditional RNN, LSTM can process a longer sequence of voice data through the combination of three kinds of controllers and achieve better voice recognition. LSTM has shown state-of-the-art performance on many tasks of speech recognition (23–25). To improve the performance further, we used a variant of LSTM, which is known as Stacked maxout LSTMs (26). The maxout LSTMs architecture is illustrated in Figure 4. In the present study, three maxout LSTM layers were stacked to build the Stacked maxout LSTMs.

Maxout units can summarize a group of spatially neighboring neurons in a lower layer that is capable of achieving the property of local translation invariance. Specifically, output *h*_*t*_ from the lower maxout LSTM layer is the input *x*_*t*_ of the upper maxout LSTM layer. These Stacked maxout LSTMs networks have the power to combine the multiple levels or representations with flexible use of long-range context. The equations of the maxout LSTM layers are as follows:


(1) $$\begin{array}{r} i_{t}=\sigma \left(W_{xi}x_{t}+W_{hi}h_{t-1}+W_{ci}c_{t-1}+b_{i}\right) \end{array}$$

(2) $$\begin{array}{r} f_{t}=\sigma \left(W_{xf}x_{t}+W_{hf}h_{t-1}+W_{cf}c_{t-1}+b_{f}\right) \end{array}$$

(3) $$\begin{array}{r} a_{t}=\max_{i=1}^{G}\left(W_{xci}x_{t}+W_{hci}h_{t-1}+b_{ci}\right) \end{array}$$

(4) $$\begin{array}{r} c_{t}=f_{t}c_{t-1}+i_{t}a_{t} \end{array}$$

(5) $$\begin{array}{r} o_{t}=\sigma \left(W_{xo}x_{t}+W_{ho}h_{t-1}+W_{co}c_{t}+b_{o}\right) \end{array}$$

(6) $$\begin{array}{r} h_{t}=o_{t}\tanh\left(c_{t}\right) \end{array}$$

For the equations, σ is the logistic sigmoid function, and *i, f, o, a*, and *c* are the input gate, forget gate, output gate, cell input activation, and cell state vectors, respectively, all of which are the same size as the hidden vector *h*. *W*_*ci*_, *W*_*cf*_, and *W*_*co*_ are diagonal weight matrices for peephole connections, G is the group size in the maxout unit.

#### Prosody Recognition

Dividing prosodic structure based on speech and text information, such as accent classification of prosodic words, pitch analysis of phrases at the boundary of prosodic phrases, and classification of tones at the boundary of intonation phrases.

#### Syntax Analysis

Based on text recognition, text phrase structure classification, short sentence type classification, and sentence category classification are performed.

#### Semantic Analysis

The information structure of phrases, the semantic inheritance relationship among dialogue rounds, and the topic of dialogue segments are analyzed.

#### Pragmatic Analysis

Classifying the speech act verbs firstly, and then categorizing the response type of this round of dialogue and confirming whether it is the trigger or response. In the wake of the classification, the adjacent pair category to which this round of dialogue belongs is investigated, and according to the source of the utterance, sentence category, or information structure, the corresponding answer type is determined.

#### Logical Analysis

Based on the results of the pragmatics analysis and combined with the current logical nodes, the next logical node can be determined, and through speech synthesis technology, a new round of dialogue can be pushed to users.

### System Operation Procedure Design

**Figure 5:**
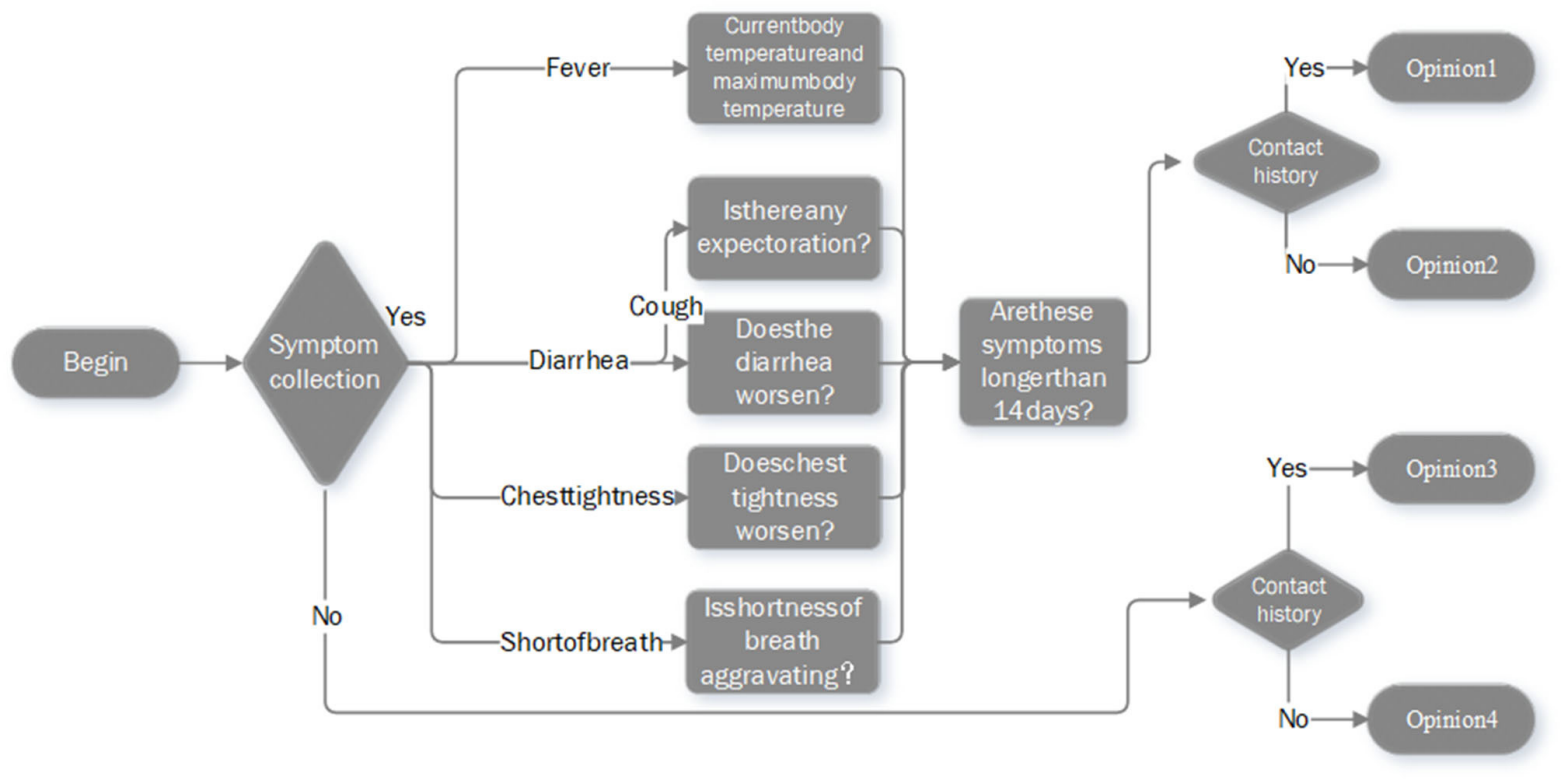
Operation procedure of the intelligent response system.

According to the actual experience of prevention, diagnosis, and treatment of COVID-19, and considering the guidance documents of the National Health Commission (27), this study designed the operation procedure of the intelligent response system (Figure 5). Firstly, when the phone call is connected, the system will first introduce itself briefly and then collect relevant symptoms information of the user. Secondly, during the conversation, the system would recognize the keywords said by the user in real time and submit the corresponding questions in a targeted manner based on the actual situation of the user. Finally, the system will provide specific COVID-19 prevention and control measures for users depending on her/his epidemiological history.

In this system, the epidemiological history includes four types. (1) People who have traveled or contacted history of the outbreak epicenters; (2) people who have been in contact with emerging epidemic areas announced by the government; (3) people who had close contact with suspected or confirmed COVID-19 patient(s); and (4) many people around occurred symptoms, such as fever, fatigue, cough, and sore throat. After completing the consultation, the system will automatically select an appropriate opinion from the four predefined guidelines to recommend to the user. The details of the guidance opinions are summarized in Table 1. In the remainder of this paper, both qualitative (system construction) and quantitative (system application) data from the real-world healthcare service of the system were collected and analyzed retrospectively.

**Table 1:** Details of the predefined guidance opinions.

**No**.	**Guidance opinion**
1	Go to the fever clinic immediately and wear a face mask for protection. Wear medical surgical masks or N95 respirator and avoid taking public transportation
2	Monitor body temperature continuously at home, take medicines for colds properly, pay attention to hand hygiene, and drink more water. If the body temperature is higher than 38°C or the discomfort symptoms exacerbate, please visit to hospital in-person for further diagnosis and treatment immediately
3	① The whole family and close contacts should be self-isolated at home for observation more than 2 weeks. Wearing face masks to communicate at home is recommended. If possible, please try to live alone or in a well-ventilated single room ② Please take more rest and drink more water, pay attention to hand hygiene and disinfection of daily necessities. If you have fever and symptoms of respiratory infection, you need to go to the designated hospital immediately
4	Please wear a face mask, wash hands frequently, avoid public gatherings, and reduce unnecessary outings

## Results

The system has been developed and put into operation since March 31, 2020. People in China can access the intelligent response system by dialing the telephone number 0371-96299.

### System Functions

#### Remote Deployment and Operation

With the support of necessary hardware and software environment conditions, this system can be deployed remotely, docking with local telephone lines, and then provides voice consultation services. During the operation of the system, the status of the system can be monitored remotely, and the consultation process mentioned above can also be adjusted remotely, which can avoid on-site operation and maintenance, reduce human contact, and decrease potential exposure of COVID-19.

#### Fast Operation Procedure Adjustment

The operation procedure of this system can be adjusted quickly and efficiently according to the actual needs, and nodes can be set up based on the original process. Functional modules, such as manual transfer and SMS distribution, can be added in if required, and the remote deployment of each procedure can be completed smoothly.

#### Multi-Dimensional Statistical Report Capability

The system can realize the statistical analysis of disconnection reasons, the dialogue data, consultation time, and geographical distribution of users. Based on the analysis, administrators can promptly identify and adjust the current existing or newly emerging problems of the system, which can help medical staff understand and count data in relation to COVID-19 well and then take more effective countermeasures.

#### Effective Protection of User Privacy and Information Security

The security module is located in the basic service layer of the system, which includes the following functions: (1) metadata management: viewing and modifying the information, such as the type, description, security level, and operation authority of each field in the data table; (2) account management: through unified management of user accounts, to make the granularity of permission control as small as possible, meanwhile, set a validity period for the permissions, and automatically recover the permissions when they expire; and (3) log management: recording and auditing the logs of account management operation, permission approval, and data access operation. Based on these functions, the security module can help the system achieves effective protection of user privacy and information security.

### Performance Evaluation

To assess the performance of the proposed intelligent response system, 100 h of telephone voice conversation from the 12,000 h corpus were extracted as the test dataset, and the comparisons of speech recognition performances of different models were conducted. The Gaussian Mixture Models and Hidden Markov Models (GMM-HMM) model was selected as the baseline model, while the KALDI toolkit was used to train the GMM-HMM model. The Stacked LSTMs model was chosen as the state-of-the-art model, in which three conventional LSTMs were stacked, and each layer had 750 LSTM cells. In the Stacked maxout LSTMs, three maxout LSTMs were stacked, each layer had the same configurations as Stacked LSTMs. These three models were evaluated based on the same dataset.

Character Error Rate (CER) was employed to evaluate the different models. CER is a typical metric of the performance of the Chinese Speech Recognition System. Through comparing the output character sequence predicted by ASR with the correct reference character sequence, CER can be computed as:


$$\begin{array}{l} CER=\frac{S+D+I}{N} \end{array}$$

Where S, D, and I are the number of substitutions, deletions, and insertions, respectively, and N is the number of words in the reference.

In terms of the test results listed in Table 2, the performance of the Stacked LSTMs model is much better than the GMM-HMM model. By replacing the input activation units in the Stacked LSTM networks with maxout units, a 2.15% relatively CER reduction can be achieved. It is should be noted that since our test data were extracted from production environments, the same source as the data used to training models, not from standard open datasets, thus the CER values presented were relatively higher than existing studies (28–30). However, according to practical experience, CER <15% is considered acceptable.

**Table 2:** CER values of different models for speech recognition.

**Models**	**CER (%)**
GMM-HMM	18.68
Stacked LSTMs	10.28
Stacked maxout LSTMs	8.13

*CER, Character Error Rate; GMM-HMM, the Gaussian Mixture Models and Hidden Markov Models; LSTMs, long short-term memory*.

### Application Effect

**Figure 6:**
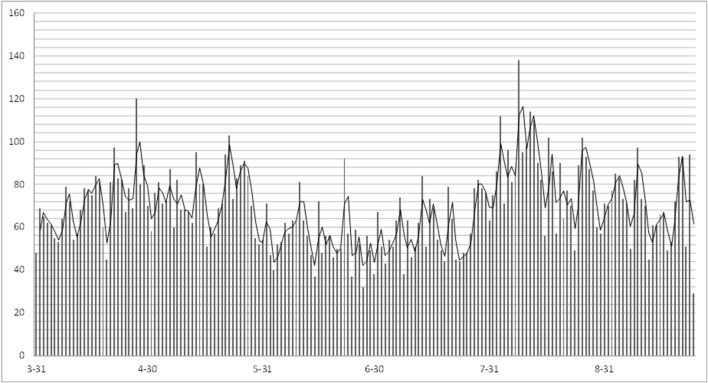
Trend chart of daily user visits.

Since the intelligent response system was launched, the average number of user visits per day was 69 (Figure 6). As of September 24, 2020, the system has received a total of 12,264 times incoming calls, among which 11,788 COVID-19-related voice consultation services were provided for the public.

**Figure 7:**
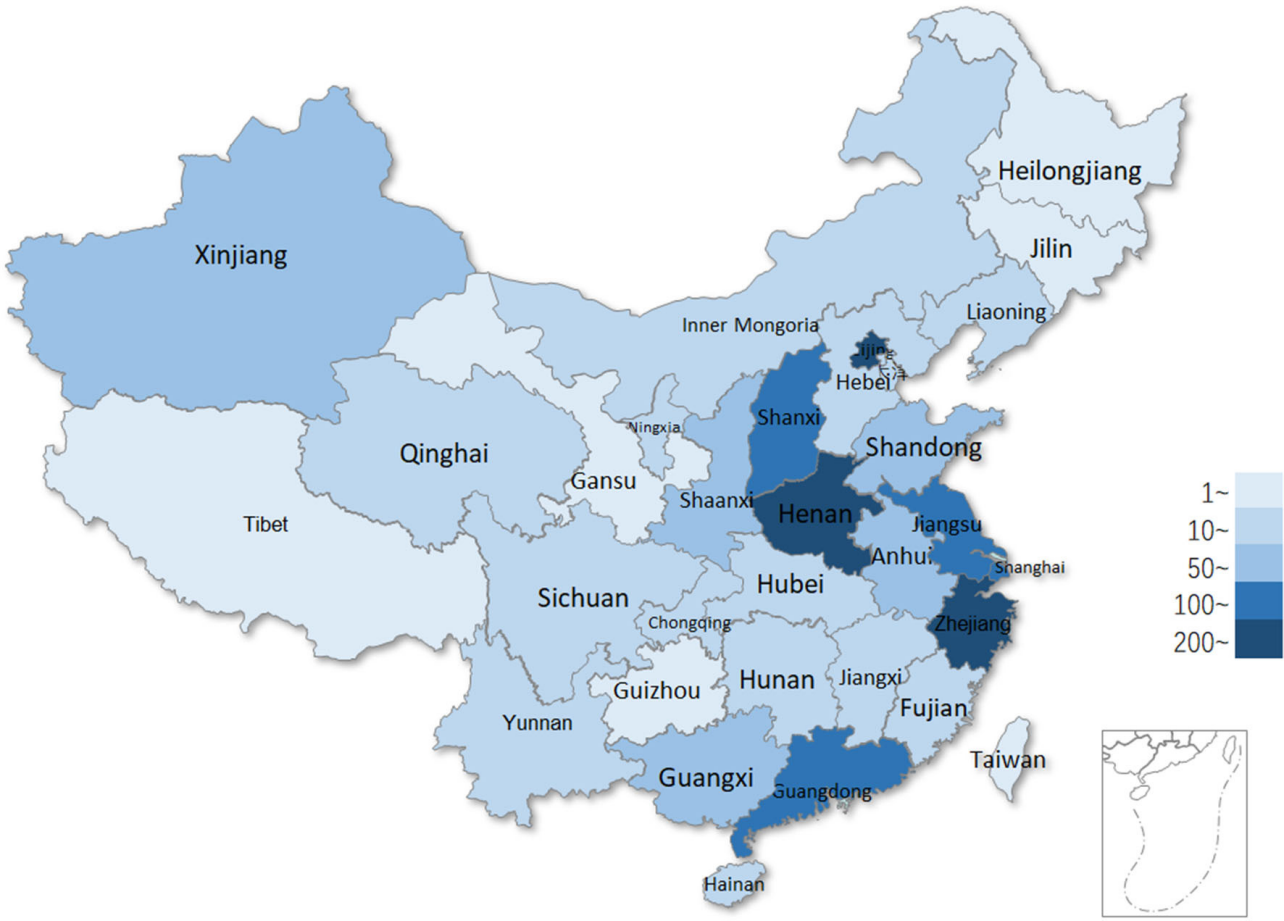
Geographical distribution of users.

**Figure 8:**
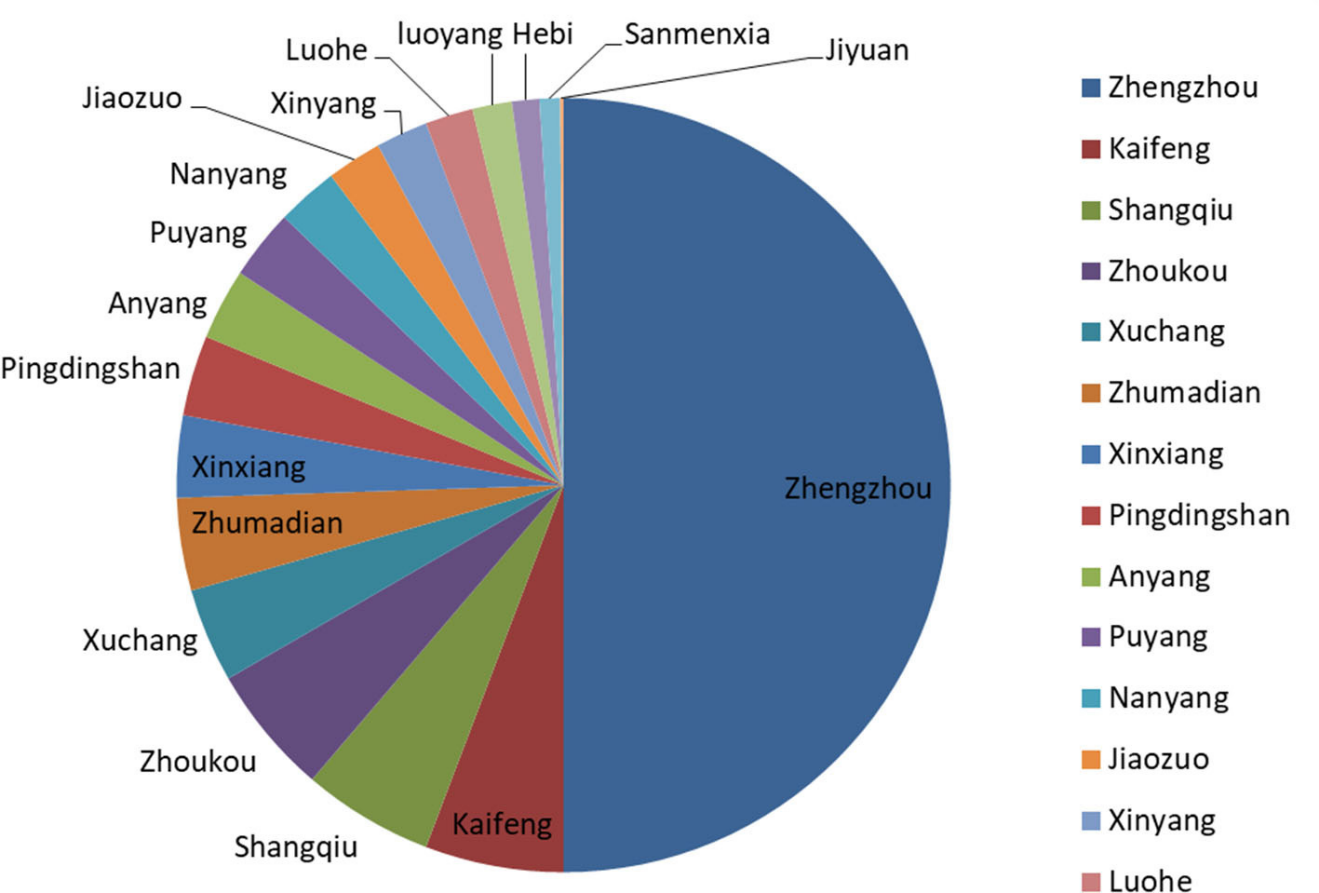
The proportion of users from the cities in Henan Province.

The geographical distribution of the incoming calls users was analyzed (Figure 7). Total 85.2% (10,054/11,788) of the users were from Henan Province and followed by Beijing, which was accounting for about 2.5% (303/11,788). In Henan Province, users from Zhengzhou city, the capital of the province, were responsible for about 50% (5,027/10,054) of the total, with the most amount (Figure 8).

**Figure 9:**
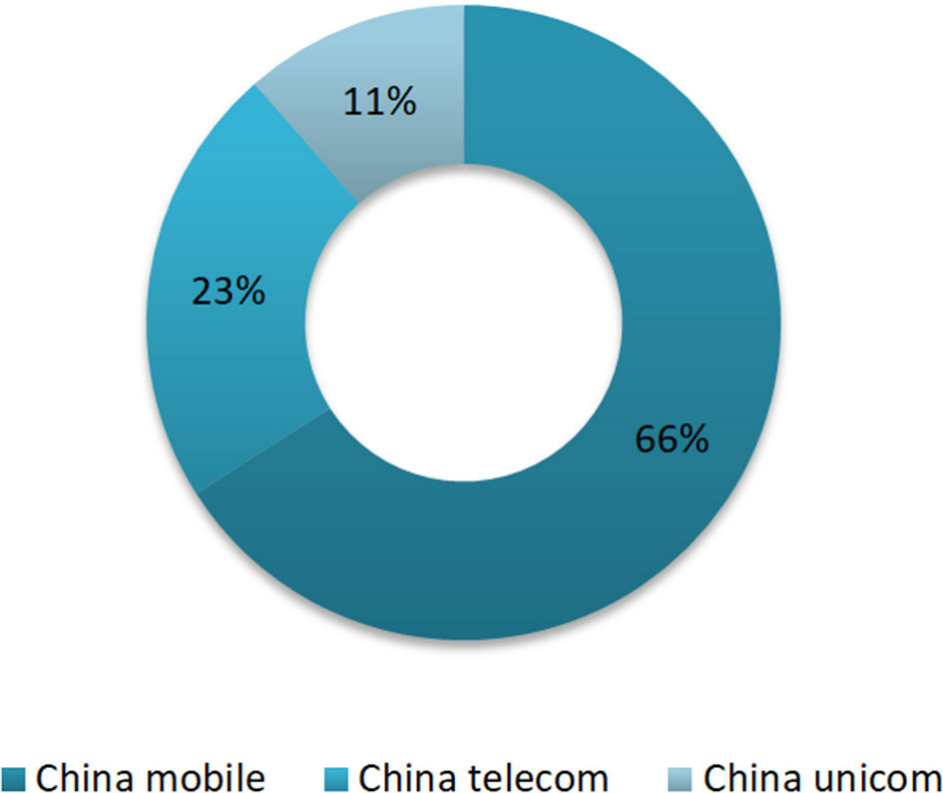
The proportion of users of mobile carriers.

The proportion of different mobile operators among users was also investigated. There are three major mobile operators in China: China Mobile, China Unicom, and China Telecom. Of all the incoming calls of the system, China Mobile with the largest proportion, and its users were responsible for 66% (7,775/11,788), while China Unicom and China Telecom were accounted for 23% (2,663/11,788) and 11% (1,350/11,788), respectively (Figure 9).

**Figure 10:**
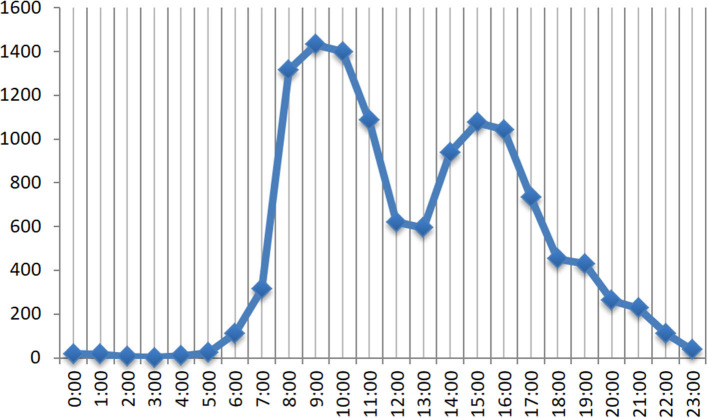
The number of incoming calls at different times of the day.

The time distribution that when users access the system for COVID-19-related voice consultation is shown in Figure 10. There was a peak period in the morning (08:00–10:00) and afternoon (14:00–16:00), respectively. Specifically, the peak time in the morning was at 09:00, and the peak in the afternoon was at 15:00.

## Discussion

Based on ASR, text to speech, and NLP technologies, the present study developed and initiated an intelligent response system for COVID-19 voice consultation. The functions, performance, and application effect of the system were then investigated. To the best of our knowledge, this is the first time, from the provincial level real-world practice in China, that the comprehensive account of an intelligent response system for COVID-19 voice consultation was explored. The findings of the current study may provide a helpful reference for further regional, national, and even international actions against the COVID-19 pandemic.

Coronavirus Diesease-2019 is a highly infectious disease, since the officially reported emergence of COVID-19 in Wuhan, China, the epidemic scale has spread rapidly, with cases arising across China and many other countries. As of February 14, 2021, in mainland China, 89,772 confirmed COVID-19 cases were reported across 31 provinces and municipalities, with 4,636 fatalities. The COVID-19 pandemic has sharply increased the demand for medical services and inevitably exceeded the maximum supply of healthcare facilities. In China, the socio-economic development gap between different regions is huge, and the distribution of medical resources is extremely uneven. For example, although 42.65% population of China lives in rural areas, ~80% of medical resources of China are concentrated in urban areas, two-thirds of which are in megacities (31, 32). This geographical inequity of access to medical resources has created relatively poor healthcare services in remote regions. Therefore, during the COVID-19 epidemic, the traditional medical services of Chinese healthcare facilities can hardly meet the needs of the public, especially in remote mountainous or rural areas (33).

The high levels of human-to-human transmission, asymptomatic infection, and long incubation period are the main reasons for the large-scale epidemic of COVID-19 (34, 35). If asymptomatic patients frequently infect others, it could vastly complicate or delay the effectiveness of prevention and control measures in response to COVID-19. Thus, measures, such as tracing and quarantining close contacts as early as possible, isolating confirmed COVID-19 cases timely, and avoiding exposure of healthy people to those infected with SARS-CoV-2 but during the asymptomatic incubation period, have been becoming effective means in the war against COVID-19 (20, 35–37). However, in the time of the COVID-19 epidemic, a large number of people visit a hospital for authoritative diagnosis and treatment, while public gatherings may increase the chance of healthy people and medical staff meeting asymptomatic COVID-19 cases, which is not conducive to effective prevention and control of COVID-19. In terms of the fact that there are no definite antiviral therapies for COVID-19 now, it is crucial to harness global efforts to take mitigation measures and emergency actions across every stage of the epidemic to contain the disease (5, 38, 39). To keep the public calm and quench unnecessary fears, healthcare facilities across the globe are expected to advise the public on what to do to stay away from COVID-19 infection, for example, advise persons experiencing symptoms of fever, dry cough, fatigue, runny nose, and anhelation to seek medical attention promptly (13, 36, 38, 39).

The intelligent response system proposed in the present study can analyze the information about consultation content of users and the response of the system. Then according to the specific situation of the users, the system dynamically provides targeted treatment opinions and action suggestions (Table 1). This may play a helpful role in screening the suspected COVID-19 cases and guiding people to stay at home for self-isolation or go to a fever clinic for further diagnosis and treatment. Healthcare consultation and medical services guidance of the intelligent response system can help to screen and triage healthy people, suspected COVID-19 cases, patients infected with SARS-CoV-2, and advise them to take preventive and control measures, such as self-isolation at home for observation, quarantine at fever clinic, or diagnosis and treatment at COVID-19 designated hospitals, respectively. It is not limited by time and space, which can realize remote triage and crowd diversion, reduce public gatherings, optimize the utilization of medical resources, improve the efficiency and coverage of healthcare services, and protect the public and medical staff from the risk of cross-infection of COVID-19.

Recurrent neural networks are networks with loops in them, allowing information to persist. LSTM is a special kind of RNNs, which can learn long-term dependencies. LSTM was firstly introduced by Hochreiter and Schmidhuber in 1997 and then was further developed and popularized by many researchers (40, 41). LSTMs work tremendously well on a large variety of problems, for many tasks, the performance of LSTM is usually better than the standard RNNs version, and almost all exciting results based on RNNs are achieved with LSTMs (41–43). In the present study, the Stacked maxout LSTMs were employed to develop the intelligent response system, for which the performance is much better than other models (with the lowest CER of 8.13%). Based on machine learning or rule-oriented dialog, intelligent conversational agents and virtual assistants, such as voice assistants and chatbots, enable communications with users via natural language, which may involve multimodal interaction support (e.g., speech, text, and sound). Generally, voice assistants typically achieve their services through a voice interface, which needs voice commands to interact and complete COVID-19-related tasks (e.g., Amazon Alexa); while chatbots primarily engage in multi-turn dialogues through text, for example as Woebot (15, 18, 44). Compared to the human-based system, the intelligent response system based on machine learning can be deployed fast and with a low hardware cost during routine operation. Due to the characteristics of accessibility, availability, and scalability for naturalistic communications with users, intelligent voice assistants have been increasingly becoming popular in the battle with the COVID-19 pandemic around the world (14, 15, 45).

In the current study, the application of the intelligent response system can achieve effective triage of outpatients, reduce public gatherings, and help control the spread of the COVID-19 epidemic. Moreover, with the help of the system, users are able to visit the nearest hospital or fever clinic according to their specific symptoms and receive appropriate diagnosis and treatment in time. These findings are consistent with other similar studies (14, 46, 47). However, compared to the voice assistants discussed in previous studies (e.g., Google Assistant, Apple Siri, and Amazon Alexa), the intelligent response system for COVID-19 voice consultation initiated in the current study has several different characteristics (14, 15, 45). First, the system only needs to be docked with the local telephone line to perform intelligent voice query services, and the response process of the newly deployed system can be adjusted according to local conditions. This is time-saving, fast, and convenient for both service providers and end-users, which is valuable during the COVID-19 pandemic. Second, when enjoying the COVID-19-related voice consultation, users do not need to download and install any client software or applications, thus, the system is relatively user-friendly with no technological proficiency requirement for users (45). Third, the deployment and application of the system are not limited by IT infrastructure, Internet access or speed, costs of hardware or software components, and locations of patients and physicians. Besides, for the operation of the system, training of healthcare professionals, nurses, and users, online assistance for patients, and alterations to integrate within the current healthcare system are not required, which can save manpower and reduce unnecessary contacts or exposure.

Our findings showed that 85.2% of the users are from Henan Province and followed by Beijing (2.5%), while for the time users visited the system, there was a peak period in the morning and afternoon, respectively. By analyzing the time and geographical distribution information of the calls of users, the system can summarize and conclude the characteristics and habits of users, which can help to optimize the allocation of medical resources and improve both the quality of healthcare services and user satisfaction. For instance, 8:00–10:00 am and 2:00–4:00 pm are the peak periods for user visits (Figure 10), during this period, healthcare facilities can meet urgent healthcare consultation needs of users through provisionally increasing service capabilities and efficiency of the intelligent response system.

### Strengths and Limitations

As one of the latest applications of artificial intelligence and ICTs in the battle with the COVID-19 epidemic, the intelligent response system for COVID-19 voice consultation proposed in the current study has both social and economic advantages. First, the system can triage and divert relevant healthcare needs of the public, thereby, alleviating the already shortage of medical resources. Second, the voice consultation services of the system can reduce the number of fearful people visiting the hospital, avoid frequent public gatherings in medical institutions, and decrease the exposure and infection risk of healthy individuals and medical staff. Third, based on the system, healthcare facilities can save and optimize the allocation of medical resources, and improve the efficiency, capacity, and quality of their services. Fourth, the system provides COVID-19-related consultation services through telephone and voice, which is appropriate and helpful for those who cannot read or use smartphones, and of course increases the coverage, acceptability, and adherence of the intelligent voice services. In addition, through advising healthy people to stay at home for isolation and observation, in addition to reducing unnecessary consumption of medical resources and the cost of healthcare services, the system can also help users avoid expenses due to hospital visits and related transportation and accommodation. The prevention and control of the COVID-19 pandemic are multifaceted, and the intelligent response system for COVID-19 voice consultation developed and applied in this study can undoubtedly play a helpful role in reducing public gatherings, preserving medical resources, increasing the service capacity of healthcare facilities, and eventually curbing the spread of COVID-19.

Some limitations of the intelligent response system in practical applications also need to be acknowledged. Firstly, the intelligent voice service functions of the system are relatively simple. It can only provide suggestions in relation to the prevention and control measures of COVID-19 based on the consultation and feedback information of users, such as personal precautionary practices, self-monitoring of body temperature at home, fever clinic visits, early detection and quarantine, and COVID-19-designated hospitals treatment, which may hardly meet the additional healthcare needs of the users. Secondly, the system is designed and constructed mainly according to or adapt to the COVID-19 epidemic situations of Henan Province. Thus, the functions and contents of the intelligent voice service may not be perfectly suitable for the prevention and control of COVID-19 in other regions in China. This can be seen from the fact that the healthcare inquiries of the system were mainly concentrated in Henan Province. The plausible phenomenon suggests that to increase the coverage and capacity of the intelligent response system, there is a need to further enrich and improve its services in the future, or do some adaption and adjustment works whenever necessary, according to the actual situations and policies where it is used. Lastly, based on the epidemiological characteristics and personal symptoms, this intelligent response system can complete the preliminary screening of users and then make relevant suggestions and guidance. However, the system only provides information for the reference of users, the COVID-19 prevention and control recommendations are neither belonging to the medical category nor can they replace the hospital doctor's diagnosis and treatment. Further research and practice works are urgently needed to address these limitations.

## Conclusions

Given the general susceptibility, high prevalence, and wide distribution of COVID-19 across the world, substantial and various public health intervention and control measures involving social, economic, and healthcare sectors, especially the vital response arrangements based on the application and analysis of real-world data are continuously warranted. Based on NLP and modern ICTs, the present study designed and deployed an intelligent response system for COVID-19 prevention and control. Through identifying and analyzing the voice information of users, the intelligent response system realized functions, such as user-oriented intelligent inquiry, screening of suspected COVID-19 cases, and targeted recommendations of response measures, which achieved appreciable practical application effects. To further improve the efficiency and quality of prevention, diagnosis, and treatment of COVID-19, in the future, the improvement and application of the system should take the actual medical service activities of clinicians into consideration. For instance, through integrating different function models into the system to promote its versatility and then to increase the capacity of the system in the battle with COVID-19. Generally, in terms of the unprecedented the COVID-19 pandemic, the provision of inquiry services through an intelligent response system in this study plays a valuable role in optimizing the allocation of healthcare resources, improving the efficiency of medical services, saving medical expenses, containing the new pandemic, and protecting vulnerable groups.

## Data Availability Statement

The datasets used and/or analyzed during the current study are available from the corresponding author on reasonable request.

## Author Contributions

JS, JG, JZ, and YZ conceptualized, designed, initiated the study, reviewed, and revised the manuscript. JS, JG, and YZ drafted the initial manuscript. MY, YL, XH, FC, and QM were involved in the development of methodology and discussion of the article structure. All authors have read and approved the final manuscript as submitted.

## Funding

This work was supported by the National Key R&D Program of China (Grant No. 2017YFC0909901), the Natural Science Foundation of Henan Province of China (202300410409), and the Joint Construction Project of the Henan Province Medical Science and Technology Research Plan (2018) (Grant No. 2018020120). The funders played no role in the design, development, or interpretation of the present work.

## Author Disclaimer

The views expressed in the article are those of the authors and do not necessarily reflect the position of the funding bodies.

## Conflict of Interest

The authors declare that the research was conducted in the absence of any commercial or financial relationships that could be construed as a potential conflict of interest.

## Publisher's Note

All claims expressed in this article are solely those of the authors and do not necessarily represent those of their affiliated organizations, or those of the publisher, the editors and the reviewers. Any product that may be evaluated in this article, or claim that may be made by its manufacturer, is not guaranteed or endorsed by the publisher.

## Abbreviations

COVID-19: novel coronavirus 2019
ICT: information and communications technology
ASR: automatic speech recognition
WHO: World Health Organization
NLP: natural language processing
API: application programming interface
SMS: short messaging service
LSTM: long short-term memory
RNN: recurrent neural networks
CER: character error rate.
